# Fifty years of sperm cell isolations: from structural to omic studies

**DOI:** 10.1093/jxb/erad117

**Published:** 2023-04-07

**Authors:** María Flores-Tornero, Jörg D Becker

**Affiliations:** Instituto de Tecnologia Química e Biológica, Universidade Nova de Lisboa, Av. da República, Oeiras, 2780-157, Portugal; Instituto de Tecnologia Química e Biológica, Universidade Nova de Lisboa, Av. da República, Oeiras, 2780-157, Portugal; University College Dublin Ireland

**Keywords:** Epigenomics, *in vitro* fertilization, isolation techniques, pollen, proteomics, scRNA-seq, sperm cells, transcriptomics

## Abstract

The fusion of male and female gametes is a fundamental process in the perpetuation and diversification of species. During the last 50 years, significant efforts have been made to isolate and characterize sperm cells from flowering plants, and to identify how these cells interact with female gametes to achieve double fertilization. The first techniques and analytical approaches not only provided structural and biochemical characterizations of plant sperm cells but also paved the way for *in vitro* fertilization studies. Further technological advances then led to unique insights into sperm biology at the transcriptomic, proteomic, and epigenetic level. Starting with a historical overview of sperm cell isolation techniques, we provide examples of how these contributed to create our current knowledge of sperm cell biology, and point out remaining challenges.

## Teaser

We provide a historical overview of studies involving sperm cell isolation, their methodologies, and contributions to the structure, biochemistry, *in vitro*fertilization, and omic analysis of isolated male gametes.

## Introduction

The success of angiosperm reproduction ultimately depends on the fusion of two sperm cells with either the egg or the central cell to generate the future embryo and its nurturing endospermic tissue, respectively. Hence, it is not unexpected that plant male and female gametes have been the subject of systematic studies. Those concentrating on the role of the male gametes are more numerous since their removal from pollen grains is less complex than the separation of the female gametes from the ovule tissues.

**Fig. 1.**
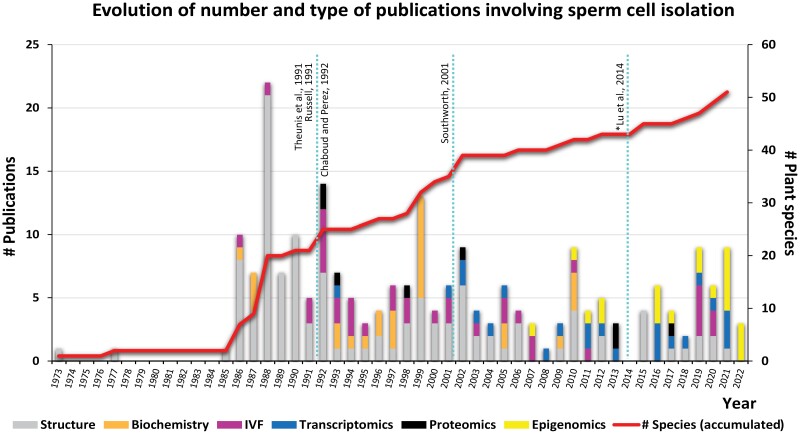
Number of publications involving sperm cell isolations during the last 50 years. Publications have been divided into six main categories according to their specific goal and the information provided (see text). This graphic shows the evolution in number of publications and the progressive shift from basic morphological descriptions to more advanced omic studies. The red line indicates the accumulative number of species whose sperm cells have been successfully isolated. The light blue dotted line indicates the most comprehensive reviews made so far. *Lu *et al*. (2014) is only available in Chinese.

Since the first *in vitro* observation of isolated sperm cells almost 50 years ago, there have been numerous publications describing different strategies to isolate these cells from several plant species and with different research goals (Fig. 1). These publications can be distributed into six main research categories, focused on the structure and biochemistry of sperm cells, their transcriptomic, proteomic, or epigenomic profiling, and their use for *in vitro* fertilization (IVF). In various studies, especially the earliest ones, the main goal was to characterize the structure of isolated sperm cells, providing information about external/internal morphology and cytoplasmic and organellar content. However, in the early 1990s there was a shift towards biochemical aspects such as cell viability and preservation, immunostainings, and the development of antibodies against molecules of the sperm cell surface. The publication of the first IVF study in *Zea mays* (Kranz *et al*., 1991b) and the successful regeneration of a mature maize plant (Kranz and Lörz, 1993) triggered the development of many other studies in the following years, involving gamete isolation in different species and the establishment of the new and promising field of IVF. Already in the new millennium, the rise of omics ­technologies slowly triggered the replacement of classical biochemical studies by transcriptomic and proteomic approaches, and, more recently, epigenomics.

An in-depth characterization of mature male gametes has been affected by biological limitations such as late pollen mitosis II in bicellular pollen grains, and methodological/technical restrictions, for example the lack of suitable isolation protocols or powerful tools for holistic analyses. During the late 1980s, approaches to tackle these problems were gradually developed, and detailed information about isolation techniques of male gametes, yield, viability, structure, and biochemistry were described in several reviews from the early 1990s (Russell, 1991; Theunis *et al*., 1991; Chaboud and Perez, 1992). Ten years later, the last and most updated review available on this topic gathered all the technological improvements in sperm cell isolation, quality and application in the emerging field of IVF, antibody development, and gene expression (Southworth, 2001). However, during the last 20 years, and especially with the advent of omics tools, our knowledge base on plant sperm cell biology has vastly increased, warranting an updated, holistic review of this topic.

Here we survey the last 50 years of sperm cell isolations and how they contributed to our current knowledge of plant sperm biology. First, we discuss the main biological issues in isolating sperm cells and summarize all isolation protocols implemented so far. Then we briefly discuss the contribution of selected publications, classified into structural, biochemical, IVF, transcriptomics, proteomics, and epigenomics studies. Studies about isolated generative cells, sperm cells inside pollen grains, or pollen tubes are beyond the scope of this review.

## Pollen types and germination techniques

**Fig. 2.**
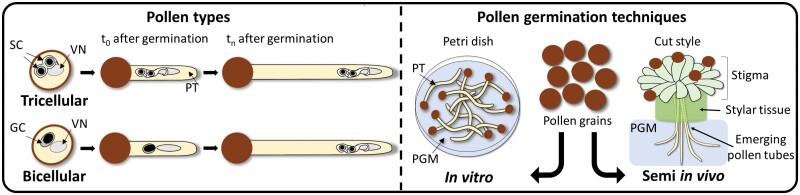
Pollen types and germination techniques. GC, generative cell; PGM, pollen germination medium; PT, pollen tube; SC, sperm cell; VN, vegetative nucleus.

Several species, including monocots such as *Oryza sativa* and *Z. mays*, or dicots such as *Brassica napus* or *Spinacea oleracea*, produce tricellular pollen grains, in which the two sperm cells and the vegetative nucleus are already formed inside the male gametophyte at anthesis (Fig. 2). From this type of pollen grains, sperm cells are obtained directly by physical or chemical procedures inside a Petri dish (*in vitro*). However, this is the situation for only 30% of flowering plants (Williams *et al*., 2014), meaning that most angiosperms release bicellular pollen at anthesis, containing a vegetative nucleus and a generative cell, with the latter only dividing into two sperm cells after pollen germination, inside the pollen tube (Fig. 2).

The time for generative cells to divide *in vitro* is species specific, ranging from 5 h after pollen germination in *Torenia fournieri* (Chen *et al*., 2006) to 32 h in *Zephyranthes candida* (Yong-sheng and Hong-yuan, 1992). Additionally, the timing is highly dependent on the temperature and specific components in the germination media (e.g. reduced forms of nitrogen or polyethylene glycol) (Read *et al.*, 1993), or on the influence of female tissues such as in *Allium tuberosum* (Lin *et al.*, 2020). To overcome this bottleneck, pollen grains are germinated on the stigma of a cut style, allowing the pollen tube to grow under the influence of female tissues and thus triggering generative cell division. Then, the pollen tube emerges from the cut end of the style carrying the two sperm cells, ready to be isolated *in vitro.* According to Cao *et al.* (1996), this *in vivo*–*in vitro* technique, currently known as a ‘semi *in vivo*’ system, was first developed in *Pyrus serotine* (Hiratsuka, 1982) but was later adapted to *Rhododendron* sp. and *Gladioulus gandavensis* (Shivanna *et al.*, 1988), work that popularized this useful technique (Fig. 2). Thanks to this method, sperm cells from many other bicellular pollen species such as *Iris tectorium* or *Hemerocallis minor* were characterized for the first time (Yong-sheng and Hong-yuan, 1992), overcoming one of the main biological issues for sperm cell observation.

## Sperm cell isolation procedures

**Fig. 3.**
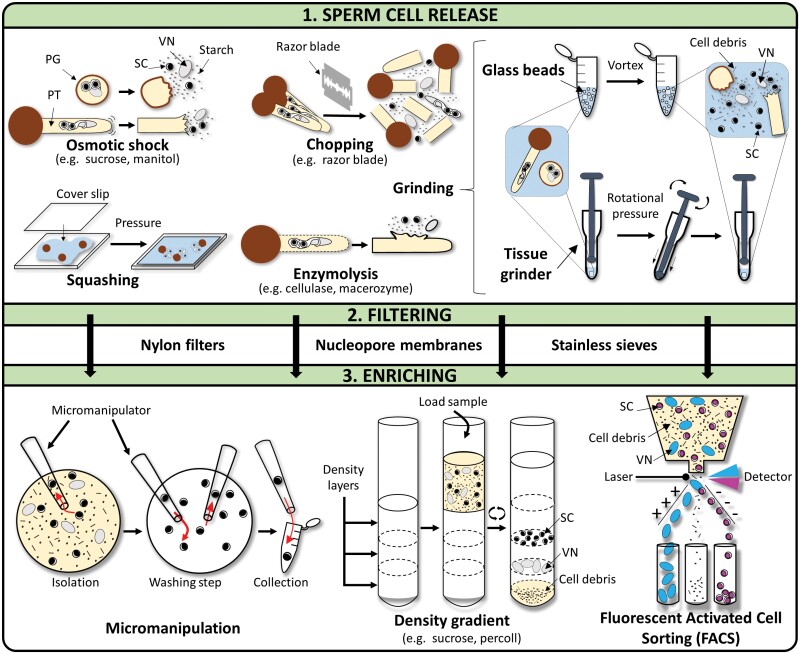
Overview of steps and methodologies for sperm cell isolation. PG, pollen grain; PT, pollen tube; SC, sperm cell; VN, vegetative nucleus.

Once sperm cells have formed, several strategies have been developed to release, filter, and enrich male gametes for further analysis (Fig. 3).

### Osmotic shock

**Fig. 4.**
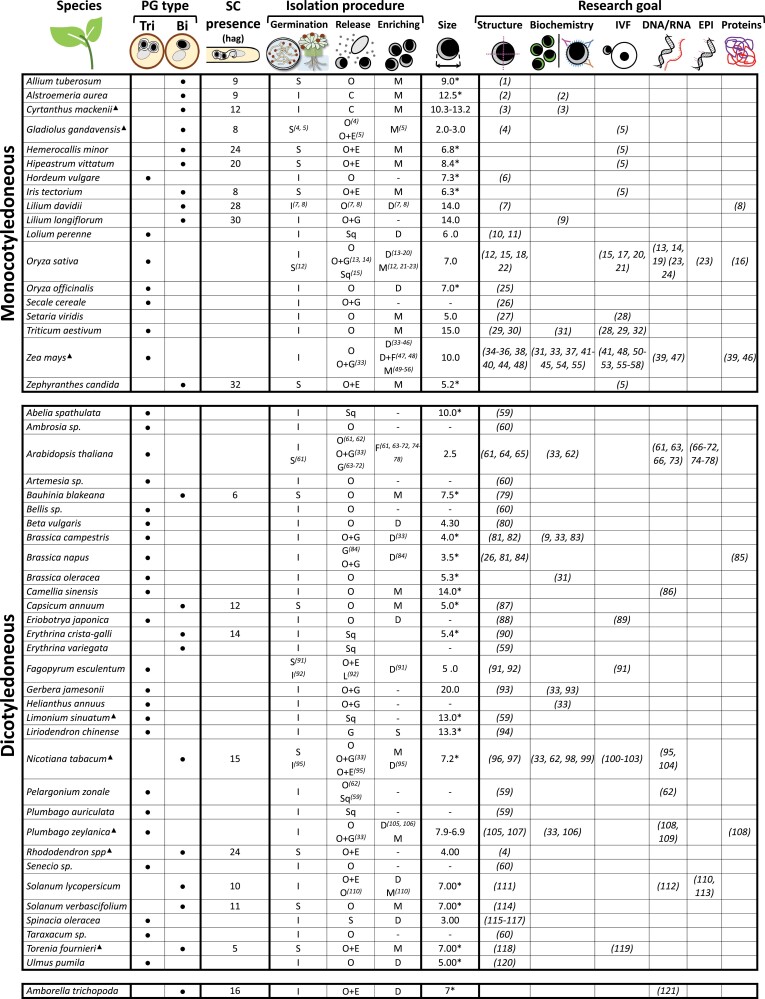
Plant species whose sperm cells have been isolated from living tissue for different research goals. An asterisk indicates a value calculated based on the pictures in the study. A filled triangle indicates species with any kind of reported difference between the two sperm cells, indicating the smallest and the largest size documented in the case of dimorphism. Numbers in parenthesis indicate the reference study (see reference list in Zenodo https://doi.org/10.5281/zenodo.7764167). Bi, bicellular; C, pollen tube chopping; D, density gradient; E, enzymatic digestion; F, FACS; G, grinding; hag, hours after germination; I, *In vitro*; IVF, *in vitro* fertilization; L, laser shockwave; M, micromanipulator; O, osmotic shock; S, semi *in vivo*; SC, sperm cells; Sq, squashing; Tri, tricellular. Size is provided in μm.

The first isolated sperm cells resulted from a spontaneous osmotic burst of ungerminated pollen grains from *Hordeum vulgare* in pollen germination medium (Cass, 1973). This method is based on the capacity of the pollen grain and pollen tube to keep their integrity during quick water exchanges due to differences in osmolarity with the surrounding media. This phenomenon inspired the first large-scale sperm cell isolation protocol for *Plumbago zeylanica* (Russell, 1986), and it has been adopted alone or in combination with grinding or enzymatic treatment in 40 plant species (15 monocots and 25 dicots) out of the 51 from which sperm cells have been isolated so far (Fig. 4). Basically, apart from several salts, buffers, and cell protectants (Southworth, 2001), all solutions have in common the use of different concentrations of osmotically active compound sugars. These can be sucrose or glucose, or sugar-alcohols such as mannitol. In some species, such as *Beta vulgaris*, a pre-hydration step is necessary (Nielsen and Olesen, 1988), whereas others such as *Gerbera jamesonii* or *Lilium longiflorum* require the removal of pollen surface oils (Southworth, 2001). However, there are species such as Brassica whose pollen grains do not burst just by osmotic shock (Taylor *et al.*, 1991), and therefore alternative strategies for sperm cell release were developed.

### Enzymatic

A pre-treatment with wall-degrading enzymes such as pectinase, cellulase, and pectolyase softens the cell walls of pollen grains and pollen tubes considerably (Zhou and Wu, 1990), releasing the sperm cells in several monocots such as *G. gandavensis* (Shivanna *et al.*, 1988), dicots such as *Solanum lycopersicum* (Lu *et al*., 2015), or even a basal angiosperm such as *Amborella trichopoda* (Julca *et al.*, 2021).

### Grinding

This strategy is frequently employed in combination with an osmotic shock and involves the use of a tissue homogenizer that breaks the pollen grains and releases the sperm cells into liquid media. Originally it was a narrow tube with glass walls and a teflon pestle but more recently glass beads in 1.5 ml vials (Santos *et al.*, 2017) or even electric blenders (C. Li *et al*., 2019) were implemented in *Arabidopsis thaliana* and *Oryza sativa*, respectively.

### Squashing

In this technique, pollen grains are placed on an abraded surface and sperm cells are released by the pressure applied on top of a cover glass or a glass roller. This method has been successfully used in monocots such as *Lolium perenne* (van der Maas *et al.*, 1993) and dicots such as *S. oleracea* (Theunis *et al*., 1988; Theunis and Van Went, 1989) or *G. jamesonii* (Southworth and Knox, 1989) due to the fact that sperm cells are comparably small and therefore difficult to crush by physical techniques.

### Chopping

This method has been implemented only for pollen tubes, and uses a sharp razor blade to chop the tubes and release sperm cells into a liquid medium. Despite its limited application, it has been successfully used in species with bicellular pollen such as *Alstroemeria aurea* (Hirano and Hoshino, 2009) and *Cyrtanthus mackenii* (Hirano and Hoshino, 2010).

Apart from these classical approaches, a relatively new one uses a laser-induced micro shockwave, in which a femtosecond laser in liquid medium generates a shockwave that allows cells to be manipulated with micrometric precision. However, implementation of this method has only been reported for sperm cell isolation from *Fagopyrum esculentum* (Hosokawa *et al.*, 2004).

After sperm cell release, pollen grain and pollen tube debris are removed by filtration through nylon filters, nucleopore membranes, or stainless sieves. Then, the following different enrichment methods are available, depending on the research goal.

### Micromanipulation

This was the first and most popular enrichment method; however, despite its simplicity and reliability in collecting high-quality material without contamination from surrounding cells, this method requires specific training and time investment. It is suitable for single-cell experiments, but for bulk analysis of large cell amounts, the following methods have been developed.

### Density gradients

The mixture of released sperm cells and starch granules or other remaining small debris is loaded on top of a liquid gradient to separate these components efficiently by centrifugation according to their density. Large amounts of *P. zeylanica* sperm cells were isolated by sugar gradient (Russell, 1986). Percoll gradients were used for *Z. mays* (Dupuis *et al.*, 1987) or *S. oleracea* (Theunis and Van Went, 1989), and are more popular because of their low osmotic potential, biocompatibility, and lack of cytotoxicity. The downside of this method is the substantial amount of starting material needed, limiting its applicability to species with an abundance of pollen.

### Fluorescence-activated cell sorting (FACS)

This method separates sperm cells from a mixture according to specific parameters such as fluorescence, size, and granularity. It was implemented for the first time to sort sperm cells of *Z. mays* dyed with Hoechst (Engel *et al.*, 2003) and later on *A. thaliana* sperm cells (Borges *et al.*, 2008; Santos *et al.*, 2017). However, as it requires a high amount of starting material, specific equipment and training, and usually depends on the availability of fluorescent marker lines, its adoption in other species has been limited.

By using different combinations of these methods, sperm cells from 51 plant species have been isolated and examined with different research goals (Fig. 4). In the following sections, we will survey the knowledge gathered in terms of structure and biochemistry, and their use for IVF, and transcriptomic, proteomic, or epigenomic profiling.

## Sperm cell structure

Determination of the size, shape, and organellar content of sperm cells was the main goal of many isolation studies, especially the early ones. Sperm cells isolated from monocots such as *H. vulgare* (Cass, 1973) and *Z. mays* (Dupuis *et al.*, 1987) or dicots such as *S. oleracea* (Theunis and Van Went, 1989) and *B. oleracea* (Matthys-Rochon *et al*., 1987) show shape transitions from spindle-shaped to more spherical morphology, whereas others remain elongated such as those of *G. jamesonii* (Southworth and Knox, 1989) or ellipsoidal such as those of *Rhododendron* sp. (Shivanna *et al.*, 1988). As shown in Fig. 4, the reported size of isolated sperm cells is quite variable, but there is a tendency for them to be larger in monocots than in dicots. The first ultrastructural characterization of isolated sperm cells was performed in *Z. mays* (Wagner *et al.*, 1989). Since then, most publications have implemented TEM and SEM to provide valuable insights into sperm cell internal structure, absence of cell wall, cytoplasmic organellar content, and the cell surface in species such as *S. oleracea* (Theunis, 1990), *B. napus* (Taylor *et al.*, 1991), or *L. perenne* (Van der Maas *et al.*, 1994).

Some structural studies revealed morphological differences between the two sperm cells in terms of cell volume and surface area in *Rhododendron* sp. and *G. gandavensis* (Shivanna *et al.*, 1988), *Nicotiana tabacum* (Tian *et al.*, 2001), *T. fournieri* (Chen *et al*., 2006), and the most well-known case of *P. zeylanica* (Zhang *et al*., 1998). The latter also shows a ‘cytosolic heterospermia’ (Russell, 1991), in which both sperm cells differ in the number of DNA-containing organelles, and with the plastid-rich sperm cell preferentially fusing with the egg cell (Russell, 1985). This polarized distribution has also been observed in other species such as *Limonium sinuatum* (Saito *et al.*, 2002), but its relationship to preferential fertilization has not been confirmed.

In other species, sperm cell differences are not related to cell volume but to nuclear shape and microtubule accumulation, such as *Cyrtanthus mackenii* (Hirano and Hoshino, 2010). Actually, a ‘nuclear heterospermia’ in terms of different content of B-chromosomes has been reported in *Z. mays* sperm cells (Russell, 1991). Still, in this case, its link to preferential fertilization in this species was revisited and refuted at the beginning of this century (Faure *et al.*, 2003).

Continuing advances in microscopy, cell imaging, and 3D analysis deliver structural information with a high level of accuracy. Utilizing these technologies can provide a more in-depth look into described heterospermia, or even uncover new ones that were previously undetected due to technical restraints. The biological implications of these heterospermia observations need further research since their link to preferential fertilization remains elusive.

## Biochemical studies

Checking for cell viability is fundamental after the isolation procedure. Initially, sperm cell viability was related to membrane integrity and evaluated by polar dyes such as propidium iodide or Evans blue (Gaff and Okong’O-Ogola, 1971). However, when fluorescein diacetate (FDA) (Heslop-Harrison and Heslop-Harrison, 1970) was used for the first time in sperm cells of *Brassica oleracea*, *Z. mays*, and *Triticum aestivum* (Matthys-Rochon *et al*., 1987), this viability test was widely adopted in various species because of its simplicity and accuracy. FDA enters the cells, and only viable cells contain esterases that convert it into fluorescein, a product that can be easily detected with a conventional epifluorescence microscope.

FDA was also used to evaluate cell viability after different storing procedures, such as cryopreservation of isolated *Z. mays* sperm cells (Cass and Fabi, 1988; Roeckel and Dumas, 1993), the use of galactose and MES (Zhang *et al*., 1992), calcium, magnesium, boron, or potassium (Zhang *et al*., 1995), or even vitamins E and C in *L. perenne* (van der Maas *et al.*, 1993). FDA also revealed that the viability of sperm cells isolated from frozen pollen of *Lilium davidii* is considerably reduced (Chen *et al*., 1995).

The biochemical composition of the surface of sperm cells was also explored to improve their selection and purification after being released from pollen grains or pollen tubes. The first approach was performed with monoclonal antibodies in *Brassica campestris* (Hough *et al.*, 1986). Later on, this experiment led to immunohistochemical studies involving JIM8 and JIM13 antibodies, in which arabinogalactan proteins were identified as the first epitopes on the surface of sperm cells in *Brassica campestris* and *L. longiflorum* (Southworth and Kwiatkowski, 1996; Southworth *et al.*, 1999).

The presence and distribution of lectin-binding sites on the sperm cell surface was investigated to obtain some insights into its glycolitic profile. The first protocol was developed in sperm cells of *Z. mays* and they were labelled with fluorescein isothiocyanate conjugated with *Concanavalia ensiformis* agglutinin (Sun *et al*., 2002). A similar fluorescence intensity was found in only 60% of the cells, but the fluorescence pattern was similar in some sperm cells and irregular in others, suggesting potential differences between the two sperm cells. In another study exploring lectin-binding sites, sperm cells and generative cells of *N. tabacum* were labelled with fluorescein isothiocyanate conjugated with three different agglutinins to explore the distributive pattern of their lectine-binding sites (Fang and Sun, 2005). The results revealed differences between generative cells and sperm cells, but no difference among the two sperm cells isolated from pollen tube, ruling out a putative dimorphism at the level of surface glycoprotein distribution, at least in tobacco.

Apart from the glycolitic profile, the physical properties of the sperm cell surface were also explored. The different electrophoretic mobility of each sperm cell of *N. tabacum* indicated differences in the charge of their cell surface, suggesting a potential link to preferential fertilization (Yang *et al.*, 2005).

Finally, an original study on mitochondrial inheritance used single-cell quantification and immunoelectron microscopy to quantify mtDNA in isolated sperm cells of *N. tabacum* and *A. thaliana*, revealing the presence of a regulatory system controlling this level by a so far unknown mechanism (Wang *et al.*, 2010).

Despite an increasing availability of more sophisticated biochemical protocols, sensitive inmunostainings, or accurate glycolitic profilings to explore sperm cell viability and cell surface composition in more detail, sperm cell studies have seen a large shift to omic approaches in the last 10 years (Fig. 1).

## *In vitro* fertilization experiments

The main goal of IVF experiments is the direct observation of a sperm cell fusing with the female gamete, so that molecules, proteins, or signal peptides can be identified to provide new insights. Therefore, the availability of sperm cell isolation methods has been of paramount importance to develop this specific area.

The first attempt to manipulate and transfer isolated sperm cells on the egg cell surface to trigger IVF was performed in *H. vulgare*, albeit with little success (Jensen *et al.*, 1986). Injection of sperm cell nuclei into egg and central cells was tested for the first time in *T. fournieri* and only succeeded in a few attempts (Keijzer *et al.*, 1988), whereas in *Z. mays* a DAPI staining confirmed a 14% success rate of sperm cell delivery to both central and egg cells (Matthys-Rochon *et al*., 1994). However, a breakthrough in this field was the development of an electrofusion-mediated fertilization technique using isolated gametes of *Z. mays* (Kranz *et al.*, 1991a, b, 1998), also applied in isolated gametes of *T. aestivum* (Kovács *et al*., 1994, 1995), *T. fournieri* (Vági *et al.*, 2006), and *Setaria viridis* (D.X. Li *et al*., 2019). This technique built the base to establish an IVF system to generate mature plants from *in vitro* gamete fusions, first achieved in *Z. mays* (Kranz and Lörz, 1993), later on in *O. sativa* (Uchiumi *et al.*, 2007; Ohnishi *et al.*, 2019), and most recently in *T. aestivum* (Maryenti *et al.*, 2019). Alternatively, other chemical components have been used for gamete fusion, such as polyethylene glycol (PEG) in *N. tabacum* (Sun *et al*., 2000, 2001) or calcium chloride in *Z. mays* (Faure *et al.*, 1994). Moreover, specific sperm–egg interactions and the existence of a blocking mechanism to prevent subsequent fertilizations (polyspermia) started to be approached (Faure *et al.*, 1994; Sun *et al*., 2000). Recently, the result of multiple fertilization events has been explored in *O. sativa* (Toda *et al.*, 2016), demonstrating the viability of polyspermic plant zygotes and their ability to develop into triploid plants. Finally, this field has also been explored in species with bicellular pollen grains, where sperm cells isolated from semi *in vivo* grown pollen tubes enabled IVF experiments in several species such as *G. gandaviensis* or *Hippeastrum vittatum* (Yong-sheng and Hong-yuan, 1992).

IVF studies are traditionally very labour intensive and require considerable cell micromanipulation skills. However, one of the main handicaps is the need to isolate female gametes to interact with sperm cells for exploratory purposes. Unlike sperm cells, female gametes are deeply embedded inside the ovules, and their successful isolation has only been achieved in 18 species so far (Flores-Tornero *et al*., 2019). Additionally, the number of female gametes that can be obtained is considerably lower than in sperm cell isolations, thus being a limiting factor for IVF studies.

## Transcriptomics

Before the onset of high-throughput transcriptomic analysis, the transcriptional activity of isolated sperm cells under *in vitro* conditions was shown for the first time in *Z. mays* by detecting radioactively labelled nucleotides in the RNA from previously incubated sperm cells (Zhang *et al*., 1993; Matthys-Rochon *et al*., 1994). Later on, a cDNA library constructed from mRNA from isolated sperm cells of *O. sativa* enabled the identification of the first transcript expressed in male gametes, related to the polyubiquitin gene *Rubq1* (Gou *et al*., 2001). Another sperm cell cDNA library was created in *N. tabacum*, in which case two transcripts were identified in both generative and sperm cells, suggesting an overlapping expression profile between these male cells (Xu *et al.*, 2002). Interestingly, a polyubiquitin-encoding transcript was found only in one of the two dimorphic sperm cells of *P. zeylanica* (Singh *et al.*, 2002), providing another exciting layer of difference beyond just morphology. The diversity of mRNAs harboured in sperm cells and their relationship to transposons, plasma membrane, and secreted proteins with putative roles in gamete interactions was discovered using a cDNA library from *Z. mays* sperm cells (Engel *et al.*, 2003).

Microarrays have also been used to explore the expression profile of isolated sperm cells at a genome-wide level. The identification of male gamete promoters (Engel *et al.*, 2005) and a male gamete-specific histone AtMGH3 (Okada *et al.*, 2005) led to the creation of lines expressing sperm cell markers such as HTR10p::HTR10–mRPF (monomeric red fluorescent protein) (Ingouff *et al.*, 2007) that are widely used to visualize these cells during live-cell imaging of the double fertilization process. These fluorescence marker lines facilitated the development of the first whole-genome transcriptomic analysis of Arabidopsis male gametes and their comparison with other sporophytic tissues (Borges *et al.*, 2008). This study further confirmed the diverse transcriptomic profile of sperm cells, identifying novel mRNAs and showing a specific enrichment for DNA repair and methylation, ubiquitination, small RNA pathways, and cell cycle processes. The implementation of microarrays also helped to identify many more differentially expressed genes between the two sperm cells in *P. zeylanica*, further supporting the previous finding and encouraging the idea of a pre-targeted gamete fusion (Gou *et al.*, 2009). Genome-wide analysis of transcripts from sperm cells of *O. sativa* (Russell *et al.*, 2012) revealed their specific gene expression profile in comparison with pollen grains or seedlings. Actually, some studies have demonstrated that the transcriptomic profiles of sperm cells of different species share specific characteristics, for instance transcripts involved in chromatin condensation, or in ubiquitin-mediated proteolysis (Xin and Sun, 2010).

Limitations of microarrays, such as limited detection of low abundance transcripts or their reliance on potentially outdated genome annotations, were eventually overcome by RNA-seq methods. RNA-seq allowed the discovery not only of a highly abundant and divergent miRNA population in sperm cells of *A. thaliana* (Borges *et al.*, 2011), but also of the diversity of mRNAs created by alternative splicing (Misra *et al.*, 2022). The distribution of small RNAs across the genome was also shown in sperm cells of *O. sativa* (Li *et al*., 2020). RNA-seq transcriptomic profiling of maize sperm cells as part of a comprehensive study of zygote genome activation provided insights into histone variants and chromatin remodelling in maize male gametes (Chen *et al*., 2017). Moreover, this technique helped to identify differences in expression profile between the sperm cells, vegetative nucleus, and egg cells in *O. sativa* (Anderson *et al.*, 2013), and to assign a putative role to long non-coding RNAs in sperm cell linage development in *S. lycopersicum* (Liu *et al.*, 2018). The most comprehensive set of sperm cell transcriptome data to date can be accessed through the EVOREPRO database https://evorepro.sbs.ntu.edu.sg (Julca *et al*., 2021).

The development of FACS protocols enabled not only bulk isolation of male gametes for transcriptome analysis but also the collection of individual sperm cells to perform single-cell RNA-seq analysis (scRNA-seq) in *A. thaliana* (Misra *et al.*, 2019). Such scRNA-seq analysis promises to definitely shed light onto the long-standing debate of a putative sperm cell dimorphism at the transcriptomic level. In the last decade, a couple of studies showed that the transcriptome of pollen tubes grown *in vitro* differs from that of those grown through the pistils (Qin *et al.*, 2009; Leydon *et al.*, 2017), suggesting potential transcriptional changes in sperm cells triggered by female tissue and hypothetically related to their fertilization capacity. The question of whether those changes affect both sperm cells equally or trigger transcriptomic differences in the two sperm cells requires further research. As a first step in this direction, a recent study used FACS to sort single nuclei of sperm cells, generative cells, and vegetative nuclei at different stages of pollen development. The resulting comprehensive single-nuclei RNA-seq dataset led to the discovery that transposable element (TE) repressors MBD5, MBD6, and SILENZIO are essential for TE silencing in the vegetative nucleus (Ichino *et al*., 2022).

## Proteomics

Translational activity of sperm cells was detected for the first time in *Z. mays*, where isolated sperm cells were labelled with radioactive methionine and leucine and showed protein synthesis activity during a time course experiment (Zhang *et al*., 1993). Other studies analysed the presence of specific membrane proteins in sperm cells of *L. longiflorum* and *B. napus* (Blomstedt *et al.*, 1992), as well as in *Z. mays* by affinity chromatography (Chen *et al*., 1998). Additionally, the protein content of the plasma membrane of maize sperm cells proved to be different compared with that of somatic cells, detecting also glycoproteins with specific residues such as glucosyl and mannosyl (Xu and Tsao, 1997). Another study reported a different distribution of ubiquitinated proteins between the two sperm cells in *P. zeylanica* (Singh *et al.*, 2002), later supported by the differences observed at the transcriptomic level (Gou *et al.*, 2009).

The increasing popularity of mass spectrometry (MS) in proteomic analysis enabled the first detailed comparison between generative cells and sperm cells in *L. davidii*, unveiling specific features of sperm cell development and functional specialization (Zhao *et al.*, 2013). In addition, an interesting study on *O. sativa* was the first one using a specific number of sperm cells (3000) to identify gamete-enriched proteins by single-cell type proteomic analysis (Abiko *et al.*, 2013). Interestingly, T-DNA mutations of Arabidopsis orthologues of some of those candidates presented reproductive defects, confirming the potential of this approach to identify new molecular players involved in fertilization. With a similar approach by using multi-omics analysis in maize sperm cells, a recent study discovered two Arabidopsis sperm-specific membrane proteins, DMP8 and DMP9, involved in gamete fusion (Cyprys *et al.*, 2019; Sprunck, 2020). These sperm cell membrane proteins, together with GEX2 (Mori *et al*., 2014) and HAP2/GCS1 (Mori *et al*., 2006; von Besser *et al.*, 2006), are the essential proteins reported so far to be involved in gamete interaction.

Currently, despite all analytical and methodological improvements, proteomic studies on isolated sperm cells are still scarce (see Fig. 4). Their implementation would be beneficial to keep addressing questions related to gamete interactions, regulated by small peptide secretion or ligand–receptor protein signalling.

## Epigenomics

In the last 10 years, the isolation of pure sperm cells has been essential for exploring their epigenetic status, providing important insight into the patterns and organization of histone modifications, DNA methylation, and small RNA activity. Here we focus on studies involving the analysis of isolated sperm cells and refer the reader to other recent reviews concerning epigenetics studies during sporogenesis and seed development (Ono and Kinoshita, 2021; Tirot and Julien, 2022).

In most animal species, epigenetic reprogramming of sperm chromatin is achieved by replacing core histones with protamines, which effectively removes all histone marks, leads to chromatin compaction, and blocks transcription (Borg and Berger, 2015). Some chromatin compaction is also observed in plant sperm cells but, given that angiosperms lack protamine, this involves the sperm-expressed histone variant H2B.8 in Arabidopsis. H2B.8 mediates nuclear condensation of sperm chromatin while at the same time ensuring its transcriptional activity, in contrast to animal sperm (Buttress *et al.*, 2022). In recent years, it has been shown that paternal reprogramming establishes a sperm cell fate that is largely driven through additional changes in histone composition or their modifications. The sperm-specific histone variant H3.10 (HTR10) plays a major role in Arabidopsis sperm chromatin (Ingouff *et al.*, 2010). H3.10 is immune to K27 methylation which, combined with the activity of H3K27me3 erasers and the repression of the Polycomb complex in sperm cells, contributes to a major loss of repressive H3K27me3 marks in sperm cell chromatin and to resetting of the paternal epigenome (Borg *et al*., 2020). In contrast, chromatin analysis of the vegetative cell nucleus revealed a dramatic loss of histone H1 and H3K9me2 marks compared with sperm cell chromatin. Notably, global H3K27me3 methylation is retained in the vegetative cell nucleus, further supporting the role of H3K27me3 in pollen cell fate determination. Recently, this hypothesis was confirmed by triggering sperm cell fate in the vegetative cell nucleus through the expression of H3K27 demethylases that removed H3K27me3 methylation patterns (Huang and Sun, 2022). The observation that not all vegetative nuclei changed their cell fate suggests that additional unexplored epigenetic mechanisms might be involved in determining these two cell lineages.

DNA methylation profiles of isolated male gametes have been instrumental for our understanding of epigenetic inheritance. Bisulfite sequencing of FACS-isolated Arabidopsis sperm cells revealed high levels of CG/CHG methylation and low levels of CHH methylation, which differs substantially from what can be found in the vegetative cell nucleus. These differences could be linked to the activity of the chromatin remodeller DDM1 and the DNA methyltransferase MET1, which are both largely restricted to sperm cells (Calarco *et al.*, 2012; Ibarra *et al.*, 2012). Similar differences were reported in sperm cells and vegetative cell nuclei of *S. lycopersicum* (Lu *et al*., 2021) and *O. sativa* (Kim *et al.*, 2019; Zhou *et al.*, 2021). DNA glycosylases play a central role in establishing these differences, as has been shown for Arabidopsis and rice (Schoft *et al.*, 2011; Ibarra *et al.*, 2012; Kim *et al.*, 2019; Zhou *et al.*, 2021). In the vegetative cell, the loss of histone H1 and H3K9me2 allows DNA glycosylases to access and demethylate loci normally silenced by constitutive heterochromatin in somatic tissues (He *et al.*, 2019). These demethylated regions gain chromatin accessibility as a result, presumably through the binding of transcription factors that activate neighbouring genes that are required for pollen tube growth (Borg *et al.*, 2021; Khouider *et al.*, 2021). It is also the activity of these DNA glycosylases that leads to the resetting of epigenetic marks induced by hyperosmotic stress, thus restricting the transmission of this stress memory to the female germline (Wibowo *et al.*, 2016). Thus, the reprogramming of repressive epigenetic marks, namely H3K27me3 in sperm cells and DNA-H3K9 methylation in the vegetative cell, appears to act at the crux of cell fate decisions required during the plant life cycle transitions (Vigneau and Borg, 2021).

The hypomethylation of chromatin in the vegetative cell nucleus also enables the expression of TEs that produce 21 nt to 22 nt siRNAs. Interestingly, it has been proposed that these siRNAs might be transferred to the sperm cells where they reinforce TE silencing via the RNA-directed DNA methylation (RdDM) pathway (Slotkin *et al.*, 2009; Martínez *et al.*, 2016), although the precise mechanism of this transport remains to be elucidated. These findings emphasize the important relationship within the so-called ‘Male Germ Unit’ (Dumas *et al.*, 1985), describing the physical connection between the sperm cells and the vegetative nucleus. Although the potential communication between the vegetative nucleus and sperm cells has been the focus of several studies (McCue *et al.*, 2011), the true extent of such a communication link and its biological implications remain to be established.

## Concluding remarks and future perspectives

During the last 20 years of research on the biology of plant sperm cells, early bottlenecks in isolation methods, cell yield, and purity issues were finally overcome. These improvements were essential to avoid contaminations from neighbouring cells and ensure the quality of downstream omic analyses, undeniably expanding our understanding of sperm cell biology across plant species and their central role in plant reproduction.

These new approaches will clarify the long-standing debate of heterospermia, its biological function across plant species, and, more importantly, its putative link to preferential fertilization. Such confirmation would have unprecedented biotechnological implications, including but not limited to, a targeted delivery of specific components to each female gamete after sperm cell fusion.

Notwithstanding, omic analyses are already aiding in the detection and identification of concealed molecular factors that play important roles in gamete viability and interactions. Here, molecules involved in hybridization barriers or those that protect sperm cells from high temperatures are of special interest for applied research.

A very promising albeit challenging perspective is the holistic analysis at single-cell resolution currently emerging also in plant sciences (Mo and Jiao, 2022). The considerable improvement of new MS technologies is enabling not only proteomic analysis of bulk single-cell type collections but even true single-cell metabolomic analysis in the plant field (Guo *et al.*, 2021), as it is known that the specific metabolism of a given cell is essential to regulate important functions such as stomatal closure or C_4_ metabolism (de Souza *et al.*, 2020). Similarly, it is reasonable to think that the metabolic profile of sperm cells could also be relevant to study the double fertilization mechanism from a perspective never seen before.

Further refinements of isolation methods for plant sperm cells, in particular for crops, in combination with improvements of omics methods down to single-cell level, will eventually lead to a holistic characterization of male gametes across species. In turn, a more detailed understanding of their biology holds the promise of unlocking sperm cell engineering as a new tool for plant biotechnology.

## Data Availability

The references cited in Fig. 4 are available at Zenodo (https://doi.org/10.5281/zenodo.7764167) (Flores-Tornero and Becker, 2023).
